# Ground beetles (Coleoptera, Carabidae) of the Hanford Nuclear Site in south-central Washington State

**DOI:** 10.3897/zookeys.396.6936

**Published:** 2014-04-02

**Authors:** Chris Looney, Richard S. Zack, James R. LaBonte

**Affiliations:** 1 Washington State Department of Agriculture, 1111 Washington St. SE, Olympia WA, 98502; 2 Department of Entomology, Washington State University, Pullman, WA 99164-6382; 3 Oregon Department of Agriculture, Plant Division, 635 Capitol Street NE, Salem, OR, 97301-2532

**Keywords:** Shrub-steppe, DOE, U.S. Department of Energy, pitfall trapping

## Abstract

In this paper we report on ground beetles (Coleoptera: Carabidae) collected from the Hanford Nuclear Reservation and Hanford National Monument (together the Hanford Site), which is located in south-central Washington State. The Site is a relatively undisturbed relict of the shrub-steppe habitat present throughout much of the western Columbia Basin before the westward expansion of the United States. Species, localities, months of capture, and capture method are reported for field work conducted between 1994 and 2002. Most species were collected using pitfall traps, although other capture methods were employed. Trapping results indicate the Hanford Site supports a diverse ground beetle community, with over 90% of the 92 species captured native to North America. Four species collected during the study period are newly recorded for Washington State: *Bembidion diligens* Casey, *Calosoma obsoletum* Say, *Pseudaptinus rufulus* (LeConte), and *Stenolophus lineola* (Fabricius). Based on these data, the Site maintains a diverse ground beetle fauna and, due to its size and diversity of habitats, is an important repository of shrub-steppe biodiversity.

## Introduction

Incidental conservation on government-managed land has become an important component of biodiversity conservation in the United States, particularly on defense-related properties (Boice 2006, Stein et al. 2008). This includes Department of Energy (DOE) properties, which have protected vast tracts of lands in ecosystems that have otherwise been almost completely modified by human activity. DOE sites, though disturbed, have conserved places with high ecological and conservation value, primarily via the vast buffer areas that surround active waste storage or fuel production sites (Brown 1998, Burger 2000). Indeed, ecological research and conservation may be the most valuable legacy of the DOE properties (Dale and Parr 1998). Several large DOE holdings are managed as National Environmental Research Parks in recognition of the biodiversity and ecological value of these properties. The Hanford Site in south central Washington State is a prominent example of accidental preservation of a rare ecosystem and subsequent management for its ecological value.

**Figure 1.**
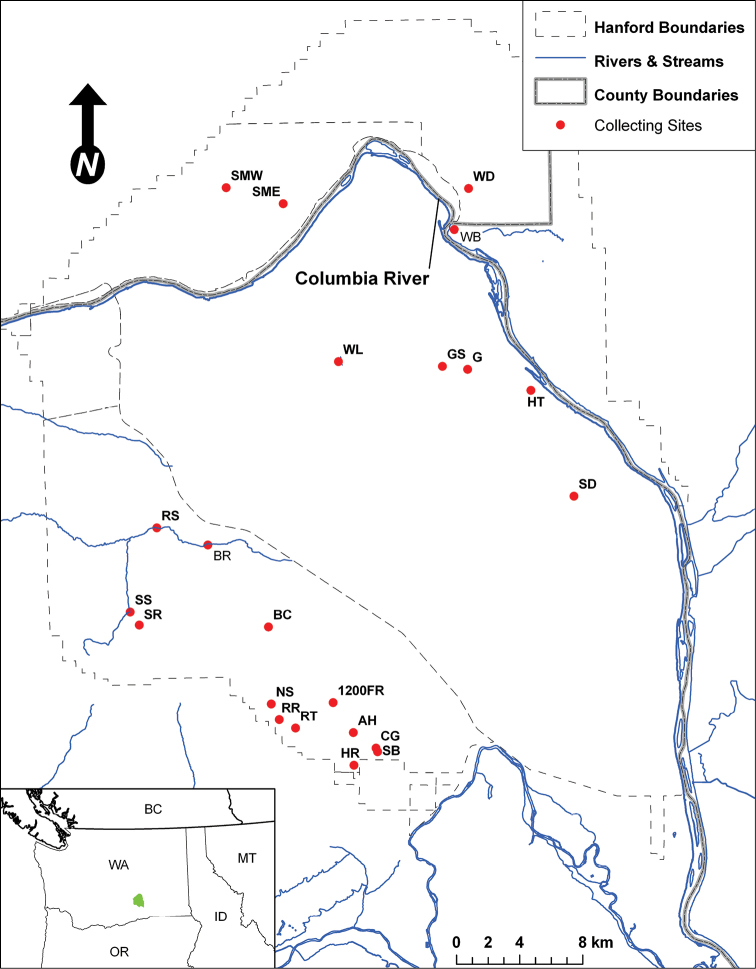
Map of the Hanford Site and collection localities.

During the past century of human activity and development much of the Columbia Basin shrub-steppe ecosystem has been converted to shrub-free grasslands and irrigated agriculture (Vale 1974), degraded from over-grazing (Jones 2000), subjected to habitat fragmentation (Welch 2005), and impacted by invasive species (Mack 1981, Knapp 1996). These changes have altered fundamental ecosystem processes and biological communities, from often-overlooked biotic soil crusts (Belnap and Phillips 2001, Ponzetti et al. 2007) to charismatic vertebrates (Connelly and Braun 1997, Van der Haegen 2007). Washington State considers its shrub-steppe ecosystem an at-risk ecological community in need of special, targeted conservation action (Washington Department of Natural Resources 2005). One of the largest contiguous tracts of high-quality shrub-steppe in Washington State is found on the Hanford Site, which encompasses more than 1,600 square kilometers of largely intact shrub-steppe habitat (Fig. 1).

The Site is located in the semi-arid region of Washington State, east of the Cascade Mountain Range. The Cascade rain shadow limits precipitation and drives wind patterns on the site. The sparse rainfall occurs almost entirely in the fall and winter months. Average annual precipitation at low elevations is only 16 cm, 38% of which is snowfall (Hoitink et al. 2005). Temperatures are high in the summer, among the highest recorded in Washington State. On average, 53 days per year have maximum temperatures equal to or exceeding 32 °C, and daily maxima exceeding 40 °C are frequent in summer months. The record high temperature for the site is 45 °C. Winter minimum temperatures average 0 °C between November and March, and below 0 °C in November through January. On average, 23 days per year have a maximum temperature ≤ 0 °C (Hoitink et al. 2005). Prevailing winds on site are north-westerly for all months, and high winds are associated with the few yearly thunderstorms experienced on site. Thunderstorm-associated wind speeds have been recorded at 114 km/hr (Neitzel 1996).

The Hanford Site is divided into several different administrative units. Central Hanford is managed by the United States Department of Energy for environmental remediation, research, and storage and processing of nuclear waste. South of Central Hanford is the Fitzner Eberhardt Arid Lands Ecology reserve, currently managed as part of the Hanford Reach National Monument. The national monument also includes the stretch of the Columbia River known as the Hanford Reach, active sand dunes along the river, the White Bluffs north of the Columbia River, the Saddle Mountain National Wildlife Refuge, and the Wahluke Unit Columbia Basin Wildlife Area. Habitats found within the reservation boundaries include loose sand dune fields, freshwater springs, expanses of perennial bunchgrass-dominated communities, shrublands, a lake, vernal pools, and degraded areas associated with human activity.

The Hanford Site has been closed to the public since the 1940s, when private and adjacent public property in the region was commandeered during World War II to create a nuclear research and fuel production area. The Site has a troubled legacy marked by radioactive materials contamination, massive and expensive remediation projects, and concomitant environmental and human health controversies (see Neitzel (1996) and Power (2008) for more discussion of the Site’s history). With the end of the Cold War, the importance of nuclear fuel production waned and activities on the Site shifted increasingly toward environmental restoration, research and management.

In 1992, the Nature Conservancy partnered with the U. S. Department of Energy to conduct a biological diversity survey of the Site. The results were intended to inform decision making and the future of the property. The biodiversity survey included plants, biological soil crusts, terrestrial vertebrates, and insects, and was the genesis for the studies reported here (Soll et al. 1999). Other insect surveys and research from the Hanford Site have concentrated on insects in general (Kimberling et al. 2001), darkling beetles (Coleoptera, Tenebrionidae; e.g., Rickard 1970, Rogers et al. 1978), shore flies (Diptera, Ephydridae; Zack 1998), weevils (Coleoptera, Curculionidae; O’Brien and Zack 1997), torymid wasps (Hymenoptera, Torymidae; Grissell and Zack 1996), Neuroptera (Zack et al. 1998) and insects associated with woody shrubs (Rogers 1979). This paper presents a list of ground beetles (Coleoptera, Carabidae, including Cicindelinae) collected on the Site between 1994 and 2002, adding distributional, ecological, and phenological information about a beetle family frequently used in ecological and environmental studies (e.g., Rykken et al. 1997, Purtauf et al. 2004, Prasad and Snyder 2006).

## Methods

Ground beetles were collected by various means, although most specimens and species were captured in unbaited pitfall traps. Pitfall traps consisted of 500ml deli cups (circumference 33.3 cm) using a 50:50 propylene glycol/water mixture as a preservative. Two sheet metal baffles (46 cm long, 7.5 cm high) were joined in a “+” shape and placed over each trap to increase effective diameter (after Morrill et al. 1990), and sheet metal lids (30.5 cm square) were added to help prevent vertebrate predation and flooding from precipitation. Pitfall trap transects were established at five sites south of the Columbia River in March, 1998, and maintained through June 1999 or December 1999. Two pitfall transects were installed in February 1999 at two freshwater springs, and maintained through December 1999. Four additional series of pitfall transects were established in April 2002 and maintained through March 2003, in sites north of the Columbia River. A few baited pitfall traps targeting Silphidae and Scarabaeidae were installed haphazardly across the Site in the summer of 1998 and spring of 1999. The traps were baited with opportunistically obtained animal dung or dead rodents, birds, or snakes suspended in cheese-cloth over the trap cup. Trap numbers per site and total trap days varied (Table 1). Trap samples were collected weekly at most sites, although sampling intervals in general were longer during winter 1999, and administrative closures at some sites occasionally lengthened other sampling intervals.

**Table 1.** Locality information for Hanford collecting sites.

Site	Abbreviation	Coordinates	Elevation (m)	Hanford Plant Community Type	Collection methods	Pitfall Traps / Total Trap-Days
1200 Foot Road	1200FR	46°24.413'N, 119°33.253'W	375	Bluebunch Wheatgrass - Sandberg’s Bluegrass	AD, HC, MV	
ALE Headquarters	AH	46°23.370'N, 119°32.27'W	375	Bunchgrass / Cheatgrass	HC, MV, L	
Benson Ranch	BR	46°29.845'N, 119°39.36'W	189	Bunchgrass/Cheatgrass	HC	
Bobcat Canyon	BC	46°27.025'N, 119°36.41'W	465-288	Bluebunch Wheatgrass - Sandberg’s Bluegrass	HC	
Cheatgrass Stand, Rattlesnake Slope	CG	46°22.829'N, 119°31.16'W	360	Bunchgrass / Cheatgrass	PT	10 / 6420
Gable Mountain	G	46°35.745'N, 119°26.38'W	130-185	Big Sage / Sandberg’s Bluegrass	HC, CT, PT	15 / 6930
Gable Summit	GS	46°35.860'N, 119°27.63'W	325	Big Sage-Rigid Sage / Bunchgrass mosaic	PT	5 / 1735
Hodges Ranch	HR	46°22.255'N, 119°32.26'W	420	Bluebunch Wheatgrass - Sandberg’s Bluegrass	HC	
Hanford Townsite	HT	46°35.001'N, 119°23.25'W	123	Abandoned Old Agricultural Fields	HC, MV	
North Ridge Spring	NS	46°24.391'N, 119°36.31'W	965	Threetip Sagebrush / Bunchgrass Mosaic	HC	
Rattlesnake Ridge	RR	46°23.842'N, 119°35.93'W	1100	Thymeleaf Buckwheat / Sandberg’s Bluegrass	HC	
Rattlesnake Spring	RS	46°30.447'N, 119°41.88'W	210	Black Greasewood / Alkali Saltgrass	PT	5 / 1395
Radio Telescope	RT	46°23.549'N, 119°35.12'W	1100	Threetip Sagebrush / Bunchgrass Mosaic	HC	
Sagebrush Stand, Rattlesnake Slope	SB	46°22.704'N, 119°31.08'W	360	Big Sagebrush - Sandberg’s Bluegrass / Cheatgrass	PT	10 / 6420
Sand Dunes	SD	46°31.369'N, 119°21.19'W	150	Bitterbrush / Indian Ricegrass	HC, PT	15 / 6930
Saddle Mtn. East	SME	46°41.496'N, 119°35.44'W	134	Bitterbrush / Indian Ricegrass	PT	10 / 3650
Saddle Mtn. West	SMW	46°42.064'N, 119°38.27'W	156	Big Sagebrush - Sandberg’s Bluegrass / Cheatgrass	PT	10 / 3650
Snively Ranch	SR	46°27.134'N, 119°42.80'W	435	Bluebunch Wheatgrass - Sandberg’s Bluegrass	HC	
Snively Spring	SS	46°27.583'N, 119°43.24'W	380	Bluebunch Wheatgrass - Sandberg’s Bluegrass	PT	5 / 1395
White Bluffs Ferry	WB	46°40.541'N, 119°26.94'W	128	Bitterbrush / Indian Ricegrass	PT	10 / 3650
Wahluke Sand Dunes	WD	46°41.935'N, 119°26.21'W	213	Bitterbrush / Indian Ricegrass	BL, HC	
West Lake	WL	46°36.066'N, 119°32.78'W	420	Non-Riverine Wetlands & Aquatic Habitats	PT	20 / 12,300

Collection Method Abbreviations: AD = coyote dung-baited pitfall trap BL = black light trap CT = carrion-baited pitfall trap HC = hand collected L = fluorescent or incandescent lights MV = mercury vapor light trap PT = unbaited pitfall traps: number of traps indicated within parentheses

Mercury vapor lights were used between 1995–1997 to collect night-flying insects, resulting in some carabid catches. A few individuals were collected during the study period at incandescent lights on the exteriors of buildings and several specimens were simply hand-collected. Abbreviations for collecting method are found in Table 1.

Samples were cleaned and sorted at the M.T. James Entomological Collection at Washington State University. Specimens were identified to species using keys in Noonan (1991), Lindroth (1961–1969) and Hatch (1953), by comparison with voucher specimens in the James Entomological Collection at Washington State University, or identified by and compared to material in the personal collection of JRL. Species names follow Bousquet (2012). Voucher specimens are deposited in the M. T. James Entomological Collection, Washington State University, Pullman, Washington, and in the William F. Barr Entomological Collection, University of Idaho, Moscow, Idaho.

Graphs of phenology and habitat association are presented for forty-five numerically prominent species collected in pitfall traps from the seven long-term sites in 1998 and 1999. The phenology data are derived from trap catches for the five pitfall sites operating between March 1998 and February 1999, but are presented on a Jan-Dec axis for ease of reading. The data represent the total numbers by month of each species captured across these sites, and provide a simple, generalized picture of when each species was active. Total beetles captured/trap-day of those same species are also presented for the seven long-term pitfall sites.

### Locality descriptions

**Figure 2.**
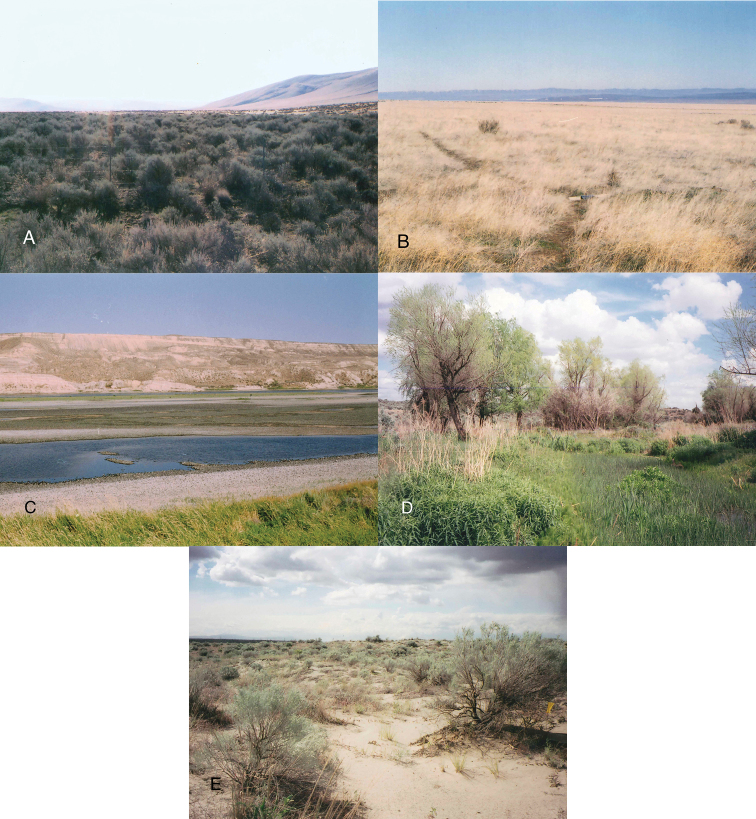
Examples of Hanford plant communities. **A** Site SB, mature sagebrush **B** Site CG, cheatgrass-dominated community **C** Site WL, alkaline pond with mixed sagebrush-cheatgrass community (sites GM and GS) in the distance **D** Site RS, freshwater spring system **E** Site SD, typical sand dune habitat.

Twenty-two collecting sites were chosen across the reserve to capture a range of environmental and biological diversity (Fig. 1, Fig. 2). The majority of the collecting sites are shrub-steppe communities, reflecting the general character of the Hanford area. Unusual habitats sampled include active sand dunes (two sites), riparian areas and springs (five sites), an alkaline pond (one site), and several significantly disturbed areas. The following list of collecting localities is organized alphabetically and briefly described. Abbreviations used to identify localities in tables and figures are in parentheses following each description. Plant species mentioned in the locality descriptions derive from on-site observations and plant lists found in Sackschewsky and Downs (2001). GPS coordinates, collecting methods, and plant community types for each site are listed in Table 1. The plant community type data are derived from a Pacific Northwest National Laboratory 2001 vegetation map; while generally indicative of habitat type, the scale of the plant community type maps is greater that of the collection sites, and some plant species typical of a mapping unit were not always present at our collecting locality. The plant community types also fail to capture important qualitative details of the different collecting sites (e.g. weediness, presence of water bodies). These factors are better related in the following descriptions.

#### 1200 Foot Road (1200FR)

The 1200 Foot Road is a dirt road running along the northern foot of Rattlesnake Ridge and is typical of the local bunchgrass and sagebrush associations.

#### ALE Headquarters (AH)

ALE headquarters is a small cluster of buildings used first by military personnel during World War II and later by research scientists. Currently the buildings are unused. Surrounding vegetation is the sagebrush/bluebunch wheatgrass (*Pseudoroegneria spicatum* (Pursh) A. Löve) type. Several colonizing weeds grow near the buildings and large open parking lots surround the complex for several hundred meters.

#### Benson Ranch (BR)

Benson Ranch was a pre-Hanford Site cattle ranch. Vegetation is primarily abandoned agricultural fields, including extensive cheatgrass (*Bromus tectorum* L.) and hedgemustard (*Sisymbrium* spp.), interspersed with bluebunch wheatgrass.

#### Bobcat Canyon (BC)

Bobcat Canyon is at the foot of north central Rattlesnake Ridge. This canyon contains a small spring system consisting of a pool only a few meters in diameter.

#### Cheatgrass Stand, Rattlesnake Slope (CG)

The road ascending Rattlesnake Ridge divides a once large sagebrush stand. Grazing and fires in the mid-1980s destroyed the north-western side of the stand, which is now composed of dense cheatgrass. This site contained no sagebrush, virtually no native shrubs, and any remaining microbiotic crust was obscured by the cheatgrass. Russian thistle (*Salsola iberica* Senne & Pau), associated with disturbed land, was also very common.

#### Gable Mountain (G)

The ENE slope of the Gable Mountain trapping site lies on a north-facing slope and is dominated by big sagebrush (*Artemisia tridentata* Nutt.), cheatgrass, and bunchgrasses in sandy soils.

#### Gable Summit (GS)

This rocky basalt outcropping has typical sagebrush/bunchgrass vegetation, and is heavily infested with cheatgrass.

#### Hanford Townsite (HT)

These remnants of the original township include crumbled foundations and abandoned roads. Vegetation is primarily introduced weeds, especially cheatgrass, with some colonizing natives (e.g., *Chrysothamnus* spp.).

#### Hodges Ranch (HR)

This area is located at the foot of the northeast slope of Rattlesnake Hills. The dominant community is bluebunch wheatgrass-Sandberg’s bluegrass with extensive patches of cheatgrass.

#### North Ridge Spring (NS)

This small, free-flowing spring lies on the northeast slope of Rattlesnake Ridge approximately 100 m below the ridge crest. The spring emerges from a concrete structure and flows for approximately 10 m downslope. Sparse riparian vegetation is present within a shrub-steppe matrix.

#### Radio Telescope (RT)

This site is part way up Rattlesnake Ridge and consists of exposed granite with thin, sparse soils and scattered vegetation.

#### Rattlesnake Ridge (RR)

Rattlesnake Ridge is an anticlinal ridge and is among the most visible features of the Hanford Site. Collections were made at or near peak elevation. This area consists of rock outcrops with thyme-leaf buckwheat (*Eriogonum thymoides* Benth.) and Sandberg’s bluegrass. Several plant species typical of ridgetops occur here, including *Phlox hoodii* Rich., *Crepis modocensis* Greene, *Balsamorhiza rosea* Nels. & Macbr., and *Salvia dorii* (Kell) Abrams.

#### Rattlesnake Spring (RS)

Rattlesnake Springs supports true riparian species, such as mature *Salix amygdaloides* Anders., *Populus trichocarpa* T. & G., and *Populus tremuloides* Michx, with extensive bulrush (*Scirpa* spp.). The spring is the largest non-alkaline water body on the site after the Columbia River, and serves as a major water source and habitat for vertebrates.

#### Sagebrush Stand, Rattlesnake Slope (SB)

The road ascending Rattlesnake Ridge divides a once large sagebrush stand. Sagebrush and Sandberg’s bluegrass dominate the southeastern side of the road, and the stand was a pristine example of mature, sage-dominated shrub-steppe. This site had the most well developed cryptogamic crust of all sampling areas, scattered native forbs and Sandberg’s bluegrass, and virtually no introduced plant species. Wildfires in 2002 destroyed the sagebrush overstory, which is now largely recovered as bunchgrass and introduced species.

#### Saddle Mountain East (SME)

This site is semi-disturbed but relatively typical shrub-steppe habitat, dominated by big sagebrush, bunchgrasses, and cheatgrass, in sandy soil, with scattered lupine and balsamroot.

#### Saddle Mountain West (SMW)

The site is semi-disturbed but relatively typical shrub-steppe habitat, dominated by big sagebrush, bunchgrasses, and cheatgrass, in sandy soil, with scattered lupine and balsamroot.

#### Sand Dunes (SD)

The sand dune field west of the Columbia River contains vast, active dunes. Vegetation is typical of active dune fields, including needle-and-thread Grass (*Stipa comata* Trin. & Rupr.) and evening primrose (*Oenothera pallida* Lindl.). Dominant shrubs include green and brown rabbitbrush (*Ericameria nauseosa* (Pall. ex Pursh) G.L. Nesom & Baird and *Chrysothamnus viscidiflorus* (Hook.) Nutt.) and bitterbrush (*Purshia tridentata* (Pursh) DC.).

#### Snively Ranch (SR)

Snively Ranch is located upstream of Snively Springs. Vegetation includes sagebrush and bluebunch wheatgrass, with extensive invasion by cheatgrass.

#### Snively Spring (SS)

This mid-elevation fresh water stream lies in the Rattlesnake Hills within a matrix of sagebrush/cheatgrass/bunchgrass. Riparian vegetation includes dense stands of nettles (*Urtica dioica* L.) and other annuals, cottonwood (*Populus* spp.), and willow (*Salix* spp.).

#### Wahluke Sand Dunes (WD)

The Wahluke sand dunes are located on the Wahluke Unit of the Hanford Reach National Monument. It is a large area of sand dunes situated north of the Columbia River. Vegetation is like that described above for the larger series of dunes located on the Hanford Site, south of the river.

#### West Lake (WL)

West Lake is the only naturally occurring lake on the Hanford Site. It is highly alkaline and surrounded by salt and alkali-tolerant vegetation (e.g., *Distichlis spicata* (L.) Greene) within the larger shrub-steppe matrix. Numerous sedge and rush species are also present, as is an extensive stand of invasive smotherweed (*Bassia hyssopifolia* (Pall.) Kuntz).

#### White Bluffs Ferry (WB)

This site is located in a shallow depression approximately 50 m from the Columbia River, near the White Bluffs Ferry landing which operated from the 1880s until the early 1940s. There are no remnants of the ferry landing or buildings. Debris litters the site, which is still used as a boat launch. Vegetation consists of scattered sagebrush in a matrix of mixed, weedy vegetation with varying amounts of cheatgrass. The soil is sandy but packed.

## Results

Ninety-two species of Carabidae were collected and identified during this study (Table 2). Eighty-six species are native to North America and the region. Six species are adventitious (indicated in Table 2 with an asterisk), all accidentally introduced from Europe (Bousquet 2012): *Agonum muelleri* (Herbst), *Amara apricaria* (Paykull), *Anisodactylus binotatus* (Fabricius), *Harpalus affinis* (Schrank), *Pterostichus melanarius melanarius* (Illiger), and *Trechus obtusus* Erichson. Most (sixty) of the species were collected only in unbaited pitfall traps and five species were collected only at mercury vapor lights. Four species were documented from Washington state for the first time (see Bousquet 2012): *Bembidion diligens* Casey, *Calosoma obsoletum* Say, *Pseudaptinus rufulus* (LeConte), and *Stenolophus lineola* (Fabricius). All these species were previously known from adjacent provinces or states (Bousquet 2012). The record for *Pseudaptinus rufulus* is the northernmost for this species (see LaBonte 1996).

**Table 2.** Carabidae species collected on the Hanford Nuclear Site between 1994 and 2002, including collecting localities, month, and method.

Species	Locality																							
	1200FR	AH	BR	BC	CG	G	GS	HR	HT	NS	RR	RS	RT	SB	SD	SME	SMW	SR	SS	WB	WD	WL	Months captured	Collecting method
*Agonoleptus conjunctus* (Say)					X																	X	V-VI	PT
*Agonum ferruginosum* (Dejean)																						X	V, XI-XII	PT
*Agonum fossiger* Dejean												X										X	III, VI, VII	PT
*Agonum melanarium* Dejean																				X			III-IV	PT
*Agonum muelleri* (Herbst)*																			X				III	PT
*Agonum placidum* (Say)		X			X							X			X				X	X		X	III-X	L, MV, PT
*Agonum suturale* (Say)												X										X	IV, VI	PT
*Agonum thoreyi* Dejean																						X	V	PT
*Amara apricaria* (Paykull)*														X								X	VII, VIII	PT
*Amara blanchardi* Hayward																						X	III-XII	PT
*Amara californica californica* Dejean					X		X					X							X			X	I-XII	PT
*Amara carinata* (LeConte)												X							X			X	III-XII	PT
*Amara confusa* LeConte	X			X	X						X				X							X	II-VII	MV
*Amara convexa* LeConte											X												V	HC
*Amara discors* Kirby					X	X								X			X					X	I, II, V, VI, IX-XII	PT
*Amara farcta* LeConte																						X	I-VIII, X	PT
*Amara littoralis* Dejean					X							X		X					X			X	IV-VIII	PT
*Amara musculis* (Say)												X			X								VIII, IX	MV
*Amara obesa* (Say)																				X			VI-X	PT
*Amara quenseli quenseli* (Schönherr)	X				X	X	X					X		X	X	X	X		X	X		X	I-XII	AD, HC, MV, PT
*Amara scitula* Zimmerman												X										X	V-VII	PT
*Anisodactylus amaroides* LeConte														X									V	PT
*Anisodactylus binotatus* (Fabricius)*																			X	X		X	IV-VIII, XI	PT
*Anisodactylus californicus* Dejean												X										X	II-X	PT
*Anisodactylus consobrinus* LeConte										X													IX	HC
*Axinopalpus biplagiatus* (Dejean)														X								X	VI, VII	PT
*Bembidion bifossulatum* (LeConte)																						X	III, VI	HC, PT
*Bembidion coloradense* Hayward																						X	III	PT
*Bembidion diligens* Casey																						X	III-IX, XI	PT
*Bembidion flohri* Bates						X																X	III-VIII, XII	CT, HC, PT
*Bembidion impotens* Casey												X							X			X	III, IV, VI	HC, PT
*Bembidion insulatum* (LeConte)																						X	III-IV	PT
*Bembidion mormon* Hayward																						X	II-XII	CT, HC, PT
*Bembidion obscurellum obscurellum* (Motschulsky)	X									X		X			X				X			X	I-XII	MV
*Bembidion obtusangulum* LeConte																						X	I-VIII, X	PT
*Bembidion patruele* Dejean																						X	III-IV, VI	PT
*Bembidion quadrimaculatum dubitans* (LeConte)															X				X			X	VI, VII, XII	HC, PT
*Bembidion rupicola* (Kirby)	X		X		X							X			X				X			X	I-IX, XI, XII	MV
*Bembidion salinarium* Casey																						X	I, III-XII	HC, PT
*Bradycellus nitidus* (Dejean)												X											VI-VIII	PT
*Bradycellus nubifer* LeConte												X							X				VI, VII, IX	PT
*Bradycellus politus* (Fall)																						X	VI-VIII	PT
*Calathus ruficollis ignicollis* Casey					X				X			X		X		X	X		X	X		X	I-XII	PT
*Calosoma cancellatum* Eschscholtz																						X	VII	PT
*Calosoma luxatum* Say	X			X	X	X	X							X	X	X	X					X	II-VIII	HC, PT
*Calosoma obsoletum* Say			X																				IV	PT
*Carabus taedatus agassii* LeConte										X									X				IV-VII	HC, PT
*Chlaenius sericeus* (Forster)																						X	V, VI, X	PT
*Chlaenius tricolor* Dejean																						X	III-VII, X, XI	PT
*Cicindela hemorrhagica hemorrhagica* LeConte																						X	VI-IX	CT, HC, PT
*Cicindela oregona oregona* LeConte																						X	IV-VIII	HC, PT
*Cicindela pugetana* Casey	X			X							X							X					III-V	HC
*Cicindela tranquebarica vibex* Horn																						X	III-V, IX, X	HC, PT
*Cylindera terricola imperfecta* (LeConte)																X						X	V-VIII	HC, PT
*Clivina oregona* Fall												X											III-V	PT
*Cymindis planipennis* LeConte	X	X			X	X	X					X		X	X	X	X		X	X		X	V-XII	AD, HC, PT
*Dicheirotrichus cognatus* (Gyllenhal)												X								X		X	I-VII, XII	PT
*Dicheirus piceus* (Ménétriés)	X	X		X	X		X	X				X		X				X	X				IV-VII, X	HC, PT
*Discoderus parallelus* (Haldeman)					X																		IV, VI	PT
*Dyschirius aratus* LeConte																						X	III-VIII, X-XII	PT
*Dyschirius politus politus* (Dejean)																						X	IV, VI, VII	PT
*Elaphrus lecontei* Crotch	X																					X	II-VI, X, XII	HC, PT
*Euryderus grossus* (Say)						X													X				VI, VII	PT
*Harpalus affinis* (Schrank)*															X					X		X	IV-VI, VIII, XI	PT
*Harpalus caliginosus* (Fabricius)																						X	VI, VII, IX	PT
*Harpalus fraternus* LeConte	X	X	X	X	X	X	X	X		X		X		X	X			X	X			X	III-IX, XII	HC, PT
*Harpalus fuscipalpis* Sturm	X		X	X	X		X			X		X	X						X			X	III, VI, VII	HC, PT
*Harpalus opacipennis* (Haldeman)																			X			X	III-VII, XI	PT
*Harpalus pensylvanicus* (DeGeer)												X			X	X						X	VII-IX	PT, HC
*Lebia viridis* Say										X									X				VII	HC, PT
*Loricera pilicornis pilicornis* (Fabricius)												X										X	III, VI, VII	HC, PT
*Microlestes linearis* (LeConte)												X										X	IV, VI-VIII	PT
*Notiophilus nitens* LeConte																			X				X	PT
*Patrobus longicornis* (Say)																				X	X	X	VI, VIII, IX	PT
*Platynus brunneomarginatus* (Mannerheim)										X		X							X			X	II-VII, XI	HC, PT
*Poecilus lucublandus* (Say)														X					X	X		X	III-XII	PT
*Poecilus scitulus* LeConte																						X	V-VII	PT
*Pseudaptinus rufulus* (LeConte)																				X			VI	PT
*Pterostichus adstrictus* Eschscholtz																						X	VI	PT
*Pterostichus corvinus* (Dejean)																				X		X	III-VIII	PT
*Pterostichus luctuosus* (Dejean)																				X		X	IV, VI, VII, XI	PT
*Pterostichus melanarius melanarius* (Illiger)*					X							X		X	X	X			X	X		X	III-XI	PT
*Rhadine jejuna* (LeConte)					X	X	X					X		X	X				X			X	I-XI	PT
*Stenolophus anceps* LeConte																					X		VI	BL
*Stenolophus comma* (Fabricius)																			X			X	III-VIII	PT
*Stenolophus fuliginosus* Dejean												X							X		X	X	IV, V, VII, VIII	PT, BL
*Stenolophus lineola* (Fabricius)																					X	X	VI-VII	PT, BL
*Stenolophus rugicollis* (LeConte)															X								VI	MV
*Syntomus americanus* (Dejean)																				X		X	IV, VI-VIII	PT
*Tachys corax* LeConte																						X	V, VIII-X	PT
*Tachys edax* LeConte																				X			IV	PT
*Trechus obtusus* Erichson*																			X	X		X	II-IV, VI, IX- X	PT
Species per locality	11	4	4	6	17	8	8	2	1	7	3	29	1	14	15	7	5	3	29	18	4	70		
Unique species per locality	0	0	1	0	1	0	0	0	0	1	1	2	0	1	1	0	0	0	2	4	1	24		

Only a few species were found in ten or more collecting sites: *Amara quenseli quenseli* (Schönherr) (twelve), *Calosoma luxatum* Say (ten), *Cymindis planipennis* LeConte (thirteen), *Dicheirus piceus* (Ménétriés) (ten), *Harpalus fraternus* LeConte (fifteen), and *Harpalus fuscipalpis* Sturm (ten) (Table 2). However, even these species had disproportionate activity/density in just one or two localities (Fig. 4). Only *Bembidion rupicola* (Kirby), *Calosoma luxatum*, *Harpalus fraternus*, and *Rhadine jejuna* (LeConte) had relatively high activity/density at three or more locales (Fig. 4). Most species were only found in or had high activity/density at a single locality, with forty species collected from only a single locality (Table 2, Fig. 4).

For comparison of fauna by site it is important to distinguish sites sampled with relatively efficient pitfall traps from those sampled opportunistically by hand or with light traps. Eleven sites have records that stem only from non pitfall-trap collections (Table 1). Twenty-three species were collected at these sites, ranging from eleven (at the 1200 Foot Road site) to one species per site (Table 2). Five species were unique to these sites, four hand-collected and one captured at a mercury vapor light. Eighty-seven species were collected at sites sampled with pitfall traps, with five to seventy species per site. West Lake had the greatest number of species (seventy), 80% of all species collected with pitfall traps. Two other localities had species counts of twenty-nine each, Rattlesnake Springs and Snively Springs. Twenty-four species were only found at the West Lake site (Table 2, Fig. 4), an order of magnitude of unique species greater than almost all other habitats. More than half (fifty-four) of the species collected with pitfall traps were captured only at riparian habitats–at one of the spring systems, White Bluffs Ferry, or West Lake.

**Figure 3.**
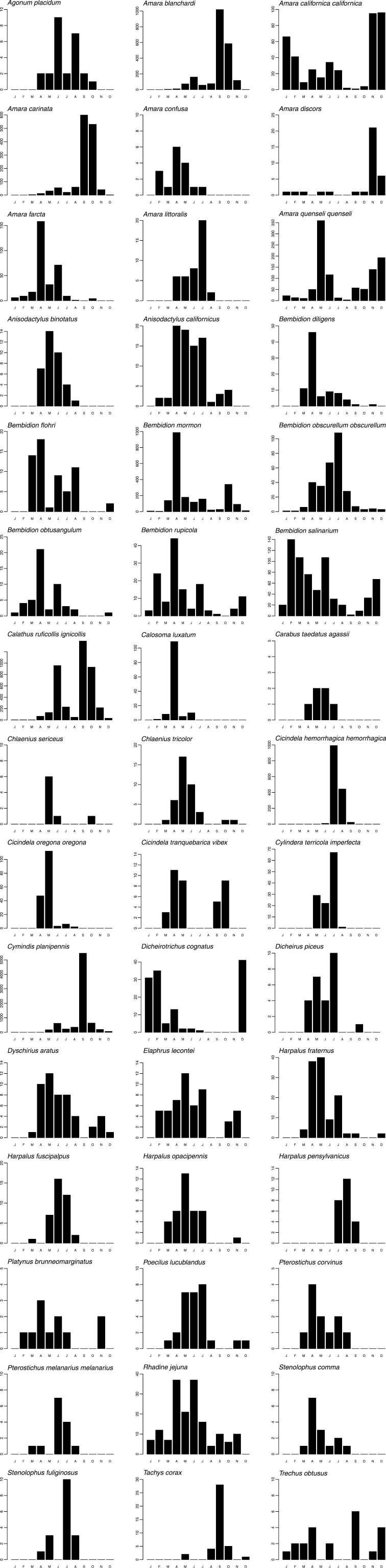
Bar graphs presenting total seasonal abundance for select pitfall-trapped carabid species. Y-axes indicate the total number captured per month, summed across all sites.

Phenologies based on activity/density were highly variable (Fig. 3). A few species were active throughout the year: *Amara californica californica* Dejean, *Amara quenseli quenseli*, *Bembidion rupicola*, *Bembidion salinarium* Casey, and *Rhadine jejuna*. Some species had very narrow peaks, with high numbers during only one or a few months, e.g., *Amara carinata* (LeConte), *Bembidion diligens*, *Calosoma luxatum*, *Chlaenius sericeus* (Forster), *Cicindela oregona oregona* LeConte, *Cymindis planipennis* LeConte, and *Tachys corax* LeConte. However, all species had distinct, and for the most part, unimodal, peaks. Although defying rigid categorization, there were some basic patterns, arbitrarily defined as: “spring-active” (March through May), e.g., *Calosoma luxatum*; “summer-active” (June through August), e.g., *Cicindela hemorrhagica hemorrhagica* LeConte; “autumn-active” (September and October), e.g., *Amara carinata*; and “winter-active” (November through February), e.g., *Amara discors* Kirby. A few species were bimodal, e.g., *Amara californica californica*, *Cicindela tranquebarica vibex* Horn, and *Trechus obtusus*.

**Figure 4.**
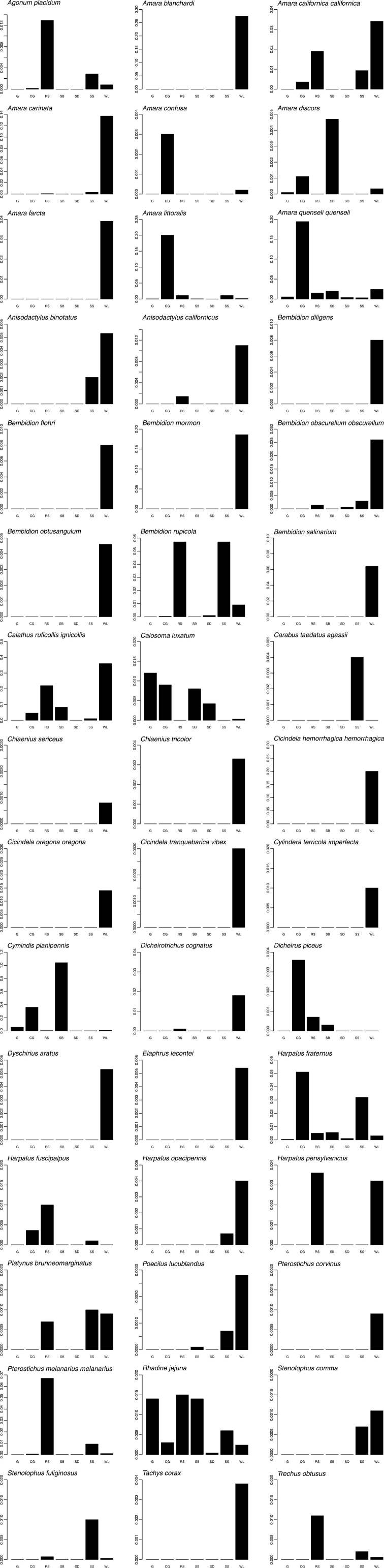
Bar graphs presenting per-trap catches of select pitfall-trapped species from seven collecting sites. Y-axis units for each graph are individuals/trap/day, over the entire collecting period. Locality abbreviations (X-axis) are found in Table 1.

## Discussion

Most, if not all, of the eighty-six indigenous species of Carabidae found at the Hanford Site in this study are typical inhabitants of shrub- and rangelands of the Columbia Basin, and the habitat data generally conform to what is known or expected from these species (Larochelle and Larivière 2003). The discovery of four carabid species previously undocumented from Washington is not surprising, particularly since many of the habitat types found at the Site have not been extensively sampled in the state. Given that there are hundreds of carabid species in adjoining British Columbia (479), Idaho (338), and Oregon (478), (Bousquet 2012), many of which are not yet known from Washington, undoubtedly further species remain to be discovered.

Data from the non-lacustrine and riparian areas of this study resemble those from other projects sampling Carabidae in the region, with the same or similar species and total number of species reported. Hampton (2005) records thirty-four carabid species (and three genera not further identified) from the Idaho National Laboratory (INL) in southeast Idaho, compiled from studies conducted between 1968 and 2001. The INL site has vegetation and soil conditions much like the Hanford Site, in that shrubs and perennial grasses typical of the Great Basin and Columbia Basin dominate the landscape. Lacustrine and riparian habitats are rare on the INL site, represented primarily by wastewater ponds, which were not specifically sampled for Carabidae (Stafford 1983, Cieminski and Flake 1995). Sampling intensity for ground beetles across the compiled studies was low, with few pitfall traps and trapping sites (Stafford 1983), although diverse collecting techniques were used overall (Stafford et al. 1986).

Blades and Maier (1996) identified thirty species of Carabidae from a single-year survey in the Okanagan Valley in southern British Columbia, also ecologically similar to this part of the Columbia Basin. That study employed a number of collecting techniques, including in-ground aluminum troughs, across six sites. One site near a fresh-water spring was sampled, although sparingly. Despite more intensive sampling, observed species richness from non-lacustrine and riparian habitats on the Hanford sites was similar (38) to richness observed in these other studies. The greater number of species reported from the Hanford Site is due to species collected only at lacustrine or riparian habitats (54), such as West Lake and the freshwater springs (Table 2).

Bodies of water provide critical and unique habitats in arid lands and are consequently biodiversity hotspots within the overall habitat matrix. These features provide more mesic conditions for species not strictly associated with water margins. For instance, of the six exotic carabid species found in this study, most were collected (three exclusively so) at the lake, river, and stream sites (Table 2). To varying degrees, these species are associated with mesic habitats (see Larochelle and Larivière 2003). Many carabid species are lacustro-riparian specialists (see Larochelle and Larivière 2003), frequently displaying surprisingly high species richness and activity/density in those settings in the Pacific Northwest and providing important trophic linkages between the aquatic and terrestrial habitats (e.g., Hering 1998, LaBonte 1998). Together, the lacustro-riparian sites contributed more than fifty species found only at those sites. The West Lake site alone had by far the greatest species richness and the greatest number of unique species (Table 2). The two spring sites were tied for second-greatest species richness, which was almost double that of any other sites (Table 2). However, those two sites shared virtually all of their lacustro-riparian species with West Lake, with only one such species, *Clivina oregona* Fall, unique between them (Table 2). The carabids associated with alkaline water bodies are predominantly lacustrine species comprising a community largely unique to the arid West. Not surprisingly, almost all of those species (*Amara blanchardi*, *Bembidion diligens*, *Bembidion flohri* Bates, *Bembidion insulatum* (LeConte), *Bembidion mormon* Hayward, *Bembidion salinarium*, *Poecilus scitulus* LeConte, and *Tachys corax* - see LaBonte 1996, Larochelle and Larivière 2003) found at the Site were collected primarily from West Lake (Table 2). This further underscores the contribution of West Lake as a critical habitat feature at the Site. The collection of several *Bembidion flohri*, otherwise found only at West Lake, in carrion-baited pitfall traps relatively far from water was an intriguing anomaly, suggesting substantial dispersal capabilities for a species existing in often widely scattered habitats.

Considering only the sites sampled with long-term pitfall traps, absence of nearby water bodies was correlated with fewer carabid species and fewer species unique to a particular site (Table 2). However, the value of the varied habitats in the Site matrix is clear as even widespread and eurytopic species such as *Harpalus fraternus* (Larochelle and Larivière 2003) displayed pronounced peaks of activity/density in at most a few locales (Fig. 4). Even the habitats collected only by hand yielded a few unique species (e.g. *Amara convexa* LeConte from site RR, *Anisodactylus consobrinus* LeConte from site HT; Table 2).

Only six of the ninety-two species recorded here are introduced and these were trapped in low numbers. This is somewhat surprising, given the long history of disturbance and human activity at the Site (Neitzel 1996, Kimberling et al. 2001). Since there are twenty-four species of introduced and established Carabidae documented from Washington (Bousquet 2012), it was expected that the introduced species component would be much greater. Furthermore, much of the habitat would seem to suitable for establishment of many of those species (Larochelle and Larivière 2003, Spence 1990). This is likely a function of isolation and reduced access to the Site, which limits introduction pathways. The function of isolation may be inferred by the even more limited introduced carabid species composition at INL, comprising only *Amara apricaria* (Stafford et al. 1986, Hampton 2005), since INL is even more remote from population centers than is the Hanford Site. It is perhaps not surprising that this species would be found at even INL since it has one of the largest distributions in North America of any introduced carabid species (Bousquet 2012). There is little documentation regarding the quality of shrub-steppe habitat and the indigenous carabid fauna versus vulnerability to introduced carabid incursion. The current paradigm is that most introduced carabid species are open habitat specialists closely associated with human disturbance (e.g., Spence 1990, Spence and Spence 1988), although some species appear to be generalists capable of invading pristine habitats (e.g., LaBonte 2011). This suggests that if introduction pathways become more pronounced it is likely the introduced carabid species component will grow, unless the xeric conditions hinder establishment.

The number and apparent abundance of indigenous versus exotic species is a crude measure of biological integrity. Past disturbance also impacts local carabid communities, notably as changes in the relative abundance of species based on their trophic habits. In a broad study of disturbed and undisturbed communities across the Hanford Site, Kimberling et al. (2001) found that species richness of polyphagous Carabidae (e.g., *Amara*, *Harpalus* – see Larochelle and Larivière 2003) increased in localities where soil disturbance or fire increased the relative proportion of weedy plant species. Sensitivity of carabids to changes in vegetation is well-known, and increased relative abundance of omnivorous or phytophagous carabid species has also been found in degraded African steppe habitats (Ouchtati et al. 2012) and simplified or weed-impacted landscapes in Europe and North America (Purtauf et al. 2005, Hansen et al. 2009).

While this study did not directly seek to evaluate changes in the carabid communities related to past disturbance, data from two adjacent pitfall trap sites provide strong evidence of such impacts to ground beetle assemblages. The CG/SB sites comprise two localities with identical soil and aspect conditions, but with a very different disturbance history. The CG site had been subject to intensive grazing and subsequent fire, and during this study was dominated extensively by the introduced grass *Bromus tectorum*. The SB site, separated from the CG site by only about twenty-eight meters, was protected from disturbance and retained a plant community rich in native species and a shrub overstory. The change in relative abundance of predatory vs. polyphagous species between these sites was dramatic, particularly visible in the relative activity/density of *Amara quenseli quenseli* and *Cymindis planipennis* (Fig. 4; see also Looney and Zack 2008).

Disturbance history varies across the site, both at and below the scale of the broadly defined sampling localities in this paper. The importance of local site variability to carabid diversity in this study is matched by the value of size of many of these community or habitat types. Quinn (2004) found that fragmentation of shrub-steppe habitat near the reservation caused subtle, yet measurable, reductions in total abundance of many arthropod groups and that species richness of predatory carabids was greater in large shrub-steppe patches than in small patches. Thus, both the complexity of habitats across the site and the vast area conserved within the site contribute to carabid biodiversity.

Seasonal activity/density peaks displayed by carabids, such as those in Fig. 3, are presumably indicative of breeding periods, at least in part (e.g., den Boer and den Boer-Daanje 1990, Thiele 1977). Carabids were previously regarded as being either spring or autumn breeders, but this is now regarded as oversimplified and it is recognized that most species cannot be so rigidly categorized (Kotze et al. 2011, den Boer and den Boer-Daanje 1990). The data in Fig. 3 appear to bear this out. Most species at the Site displayed activity/density patterns with spring or spring and summer peaks. Many of these species are known spring breeders, a behavior associated with, but not restricted to, open habitats (Larochelle and Larivière 2003).

In addition to demonstrating activity/density peaks, the data presented in Fig. 3 contribute to our knowledge of carabid seasonality in this shrub-steppe region. For most species (e.g., *Amara blanchardi*, *Amara quenseli quenseli*, *Bembidion mormon*, and *Dicheirotrichus cognatus* (Gyllenhal)), the phenology data simply expand the known activity periods (c.f. Larochelle and Larivière 2003). For a few, less well-studied species (*Dyschirius aratus* LeConte, *Elaphrus lecontei* Crotch, and *Rhadine jejuna*), the data add considerably to the known seasonality, demonstrating a much longer period of activity than was previously recorded or suggestive of a biennial lifecycle (Matalin 2007). While most species showed relatively narrow activity periods, some had surprisingly prolonged activity and were essentially active throughout the year. Most notable were those demonstrating activity/density peaks in winter (*Amara californica californica*, *Amara discors* Kirby, and *Bembidion salinarium*). Winter can be harsh at the Site, with average daily minimum temperatures at or below freezing for much of December-February (Hoitink et al. 2005). Poikilothermic insects, presumably including most if not all of the Site carabids, are normally not active when it is that cold. However, minimum temperatures are rarely below -7 °C, and frequent sunny days may allow sporadic activity peaks. Furthermore, the relatively low winter and high summer temperatures are offset by the large range between daily minimum and maximum temperatures. This difference can be as much as 8 °C in January to 17 °C in July (Hoitink et al. 2005).

These data demonstrate the biological value of the Hanford Site, deriving perhaps not so much from the presence of any particularly unique or pristine habitats, but instead from the matrix of habitats at the Site. The biological value of the Site for these insects may stem primarily from this habitat diversity, its large size, and restricted access, rather then *per se* the quality of the remaining shrub-steppe habitat. The study also emphasizes the contribution of small, local habitats to the biodiversity of the overall Site, especially with regard to water features in this arid landscape and the distinctive insect communities they support. The value of the strictly terrestrial habitats was also evident, with even widely distributed species displaying apparent habitat preference and with most species showing marked habitat fidelity. The research value of the Site was demonstrated, with significant new information provided on carabid ranges, habitat selection and activity. The Hanford Site is clearly a unique repository of the region’s natural history and a valuable resource for future research, a fact reflected in the formal designation of the Hanford Reach National Monument (Clinton 2000). As with many defense-related government properties, biological conservation has been a fortunate side-effect of the Hanford Site’s otherwise checkered past.
